# Application of the Motor Abilities Assessment as Part of a Talent Identification System in Tennis Players: A Pilot Study

**DOI:** 10.3390/ijerph19158963

**Published:** 2022-07-23

**Authors:** Jung-Piao Tsao, Chia-Che Liu, Bi-Fon Chang

**Affiliations:** 1 Department of Sports Medicine, China Medical University, Taichung City 406, Taiwan; tjp1984@mail.cmu.edu.tw; 2 Physical Education Center, Feng Chia University, Taichung City 407, Taiwan; liucc@mail.fcu.edu.tw; 3 Department of Physical Education, National Taichung University of Education, Taichung City 403, Taiwan

**Keywords:** tennis, talent identification, adaptive learning

## Abstract

In this study, we sought to develop a testing system to scientifically identify tennis talent. This testing system will provide helpful information for players who intend to pursue a professional tennis career. The experimental subjects were 18 college students consisting of 10 tennis players (including 4 soft tennis) and 8 basketball players (all males). The subjects were tested on their vertical jump, 60 m shuttle runs, and shoulder joint mobility to identify tennis talent. To statistically analyze the data, an R package was used to conduct a principal component analysis of the athletic performance indicators of the samples, and the samples were further classified via agglomerative hierarchical clustering. This study found that tennis players required more flexibility than basketball players. Regarding the differences between male and female soft tennis players, the unclassified results showed that there was a significant difference in explosive power. However, there was no significant difference in flexibility between genders. The research methods and results of this study can be used as a reference for others to build a system for identifying athletic performance characteristics in the future, and it is expected that the implementation of this system can provide sports coaches with more information for talent selection and improve the accuracy of their judgments, allowing athletes to play to their strengths.

## 1. Introduction

According to the standards set by the world’s largest technical professional organization for the advancement of technology (IEEE) on adaptive teaching, adaptive teaching utilizes artificial intelligence or data and information communication and technology (ICT) to identify students’ learning styles and learning characteristics or to diagnose learning weaknesses. The system customizes the course materials and learning path based on the student’s skills and needs [1]. Similarly, in sports, in order to place players in the appropriate positions, it is necessary to identify their athletic talents or characteristics.

Talent identification (TID) is to find young athletes with the potential to become elite athletes [2]. Currently, athletic standards in various countries are set higher, and the pressure of competition is becoming more intense. Therefore, many countries have invested millions of dollars to actively use scientific methods to find evidence of competitive advantage [3]. High-performance athletes can attract sponsors, win prize money, and have the honor of representing their country [4]. In distinguishing between giftedness and talent, Breitbach et al. argue that a gifted individual demonstrates outstanding competency in one or multiple domains of ability, and talent, in the context of a relevant environment, is “exceptional performance in one or more fields of human activity” [4]. Other studies describe talent as genetic but that success or achieving high-performance levels in the field is because one is suited to the job or activity, that is, the environment [4].

The Real Madrid football club invested one million Euros and integrated their resources to systematically find athletes with the most potential [5]. Athletes’ achievements can be traced back to environmental factors, such as training and evaluation methods [6,7]. Therefore, emphasizing the developmental process of a person’s abilities is more important than focusing on their innate gifts, and distinguishing between giftedness and ability is crucial [8]. Many studies have explored whether outstanding performance is hereditary (nature) or comes from experience and learning (nurture) [9,10,11,12]. Most scientists believe that both of these factors are significant; nevertheless, the distinction between nature and nurture remains an ongoing topic in academia [13,14].

Regardless of whether the concept of talent is correct, there is a bias toward the physical appearance in intense sports environments. For example, the selection of young athletes is usually based on their athletic physique [15]. However, Anshel and Lidor (2012) [16] argued that talent identification and development (TID) is to identify talents through systematic evaluation and feedback according to an established process. Durand-Bush and Salmela (2001) [17] pointed out that TID programs allow sports organizations to focus funding and training opportunities on athletes with the greatest potential for success. Athletic talent refers to a person with outstanding athletic ability who is above average in training, learning, and performance [18].

Current research on tennis players is limited to two groups: an elite group and a general group. All of the research results are also similar; that is, the sport-specific physical fitness of the players in the elite groups is better than that of the players in the general groups [19,20,21]. These results are expected because the physical fitness tests in all studies are specifically designed for competitive tennis. Thus, not surprisingly, the players in the elite group perform better. It is, therefore, necessary to thoroughly explore where players in the general group have problems, or whether the players in the general group are suitable for tennis.

In addition, it is also important to identify athletes whose testing results indicate they may be more suitable for tennis than their current sports. Basketball continues to be a very popular sport in Taiwan. However, due to the build of the players, Taiwan basketball is unable to be competitive at the international level. If some basketball players with the talent for tennis can be identified in childhood, perhaps tennis in Taiwan can develop more vigorously. Tennis and basketball players both rely on the support of the lower limbs to maintain balance, execute sudden changes in direction, and jump. Thus, male basketball and tennis players are compared.

This study aims to help guide players onto a more direct path to success and considers that differences between talented tennis players and general players can be assessed through their vertical jump, acceleration, and shoulder joint mobility. The speed of the vertical jump represents the player’s explosive power, which is particularly necessary during a tennis serve; a player relies on lower body strength to jump up quickly and simultaneously extend the entire body to hit the ball at the highest point of the jump in order to execute the serve at an optimal angle and speed. Moreover, assessing the vertical jump provides insight into a subject’s reaction time and movement initiation time. In tennis, the kinetic chain of each hit begins in the lower limbs, which plays a crucial role. A serve, for example, begins with knee flexion, followed by the ball toss, the swing, and jump, and finally, contact with the ball. In their studies, Chang and Liu (2013) and Chiang, Tsai, and Chiang (2015) conducted vertical jump tests to assess athletes’ lower body strength [22,23]. However, those tests could only assess the height of an athlete’s vertical jump, not the athlete’s speed during a vertical jump. In this study, the vertical jump test measures both the height and the speed of an athlete’s jump. 

In this study, the 60 m shuttle run measures the subjects’ acceleration as they sprint 20 m back and forth three consecutive times for a total distance of 60 m. With each sprint, the ability of the lower limbs can be assessed from the running form, the point of deceleration for braking, and the acceleration point. Tennis can be described as a sport involving multidirectional shuttle runs. In tennis, a shot is initiated in the lower limbs, and then the upper limbs cooperate with the explosive power of the lower limbs to execute a strong and powerful shot, which is one of the abilities that a good player needs to have. In tennis matches, the duration of a point is about 2 to 10 s, and there are about 3–4 directional changes and 4–5 strokes in between points. For each shot, tennis players run at high intensity, and the distance depends on the player’s age, level, and court surface [23,24,25].

In tennis, following the steady movement of the lower limbs, the upper limbs perform precise strokes. The shoulder joint will rotate 360 degrees forward, backward, left, and right according to the location of the target; therefore, the range of motion, flexibility, and stability of the shoulder joint are particularly important. The range of motion of the shoulder joint is mainly used to assess the muscle strength and flexibility of the upper limbs, particularly focusing on the range of internal and external rotation of the shoulder joint. Tennis players need shoulder mobility to take the racket back before hitting the ball. At the same time, the muscles surrounding the shoulder joint assist the arm in completing the action of swinging and hitting the ball. When athletes repeatedly use these muscles, strength training is necessary to prevent sports injuries [22]. Moreover, when the movement of the shoulder joint is too large or loose, the strength of the muscles around the shoulder joint is necessary to increase the stability of the joint during movement so as to avoid injury during exercise. Therefore, a certain balance needs to be maintained between the joint range of motion and muscle strength [26]. Ann et al. (2010) studied the mobility and flexibility of the shoulder joints of juvenile tennis players and found that the left and right scapulae were asymmetrical [27]. Because tennis is an overhead sport, the scapula of the dominant hand requires a larger range of motion; therefore, when both hands are in motion, the acromion of the dominant hand is higher than that of the non-dominant hand [28].

The purpose of this research is to establish a tennis talent testing system that assesses the vertical jump, 60 m shuttle run, and shoulder joint mobility. In the future, young players who intend to pursue careers in professional tennis can be advised after being evaluated by the testing system presented in this research. It is believed that this scientific identification method will effectively assist coaches, players, and parents and allow artificial intelligence to play a role in what sport an athlete chooses to specialize in.

## 2. Materials and Methods

A total of 18 college students voluntarily participated in this study conducted on 08 December 2020. The subject population comprised 7 males and 3 females who specialized in tennis, and 8 males who specialized in basketball (the average height, weight, and BMI of the 18 subjects were, respectively, 175.7 ± 2.54 cm, 74.02 ± 3.28 kg, and 23.83 ± 0.68 kg/m^2^). The two sports are among the most popular sports in Taiwan, and distinguishing unique strengths among players of these sports can benefit the effectiveness of recruitment programs. Recreational student players were not recruited in this study because distinguishing them from the athletes who train regularly does not require the methods proposed in this study. To participate in the study, the subjects had to be college students who can train normally, and exclusion criteria were sports injuries, fractures, or dislocations within the past 6 months before the study, and the subjects could not have a history of neuropathy, head trauma, or injuries in the lower extremities. Participants were fully informed of the protocol beforehand and gave their informed consent prior to the start of the study. The study strictly adhered to the approved IRB protocol. 

After discussing different international physical attribute tests and tennis conditioning exercises with a strength and conditioning coach for professional tennis players, the authors determined that the vertical jump, 60 m shuttle run, and shoulder joint mobility should be assessed for tennis talent identification. The vertical jump mainly tests the ability of players to generate strength from a half squat, and the landing is an indicator of reaction ability. In addition to testing the explosive power of the players, the 60 m shuttle run can also be used to assess the comprehensive ability of the players during repeated acceleration and deceleration. The shoulder mobility tests assess the flexibility of a player’s shoulder joint, as proper flexibility allows for more movement and acceleration of swings, which is helpful for executing serves, forehands, and backhands.

### 2.1. Tennis Talent Identification Methods

Details and methods of each test are described below.

#### 2.1.1. Counter Movement Jump

(1) The subject has a bar sensor (velocity-based training (VBT)) on their shoulders and stands in an upright posture on a force plate with feet pointing forward;
(2) The force plate measures the peak power, height, and rate of force development at 200 milliseconds (RFD200), while VBT collects the peak velocity of the concentric phase during take-off (CMJ 1);
(3) The subject quickly squats down (knees bent to a 90-degree angle) and jumps as high as possible, and the jump height is calculated (CMJ 2);
(4) Power output per kilogram of body weight is calculated (CMJ 3).

#### 2.1.2. Squat Jump

(1) The subject has a bar sensor (VBT) on their shoulders and stands in an upright posture on a force plate and squats down (knees bent to a 90-degree angle);
(2) The force plate measures the peak power, height, and rate of force development at 200 milliseconds (RFD200), while VBT collects the peak velocity of the concentric phase during take-off (SJ 1);
(3) The subject jumps vertically from a squat position with no countermovement (SJ 2);
(4) Power output per kilogram of body weight is calculated (SJ 3).

#### 2.1.3. Drop Jump

(1) The subject has a bar sensor (VBT) on their shoulders and stands on a box with a height of 30 cm;
(2) On cue, the subject steps off with one foot, lands on both feet, and immediately performs a vertical jump;
(3) In the air, knees should be straightened to 180 degrees;
(4) VBT calculates the reactive strength index (RSI) (DJ R/L).

#### 2.1.4. The 60 m Shuttle Run

(1) Cones are placed 20 m apart, and then the subject sprints back and forth for a total distance of 60 m;
(2) The subject should touch the cones each time before sprinting in the other direction;
(3) Total time is calculated as the time when the subject touches the cone after completing the third shuttle;
(4) The best time is recorded after 4 sets; the resting time between each set is 2 min (60 M).

#### 2.1.5. Shoulder Mobility: Subjects lying in Supine Position on Treatment Bed with Arms and Elbows in a 90-Degree Angle

(1) The angle of internal and external rotation of the subject’s arm is measured using a protractor (left shoulder internal rotation (LJIN 1); left shoulder external rotation (LJIT 2); right shoulder internal rotation (RJIN 1); right shoulder external rotation (RJIT 2));
(2) During the measurement of the arm’s forward and backward rotation, the subject should not shrug shoulders.

#### 2.1.6. Scapular Retraction

(1) The subject is in a kneeling prone position on the floor, and the arm being measured is extended forward, perpendicular to the floor;
(2) Subject lifts their arm as high as possible, and the height of the arm above the floor is measured with a ruler (LJIN 3, RJIT 3);
(3) During the measurement process, the subject should not bend the elbows or rotate the thoracic spine.

### 2.2. Statistical Analysis

Principal component analysis (PCA) allows the information in the multivariate data set for the 18 subjects to be visualized by reducing the dimensionality and identifying the correlated variables. By identifying the directions (or principal components), which correspond with the largest variation, the important information from the data set described by multiple quantitative variables was extracted and, in this study, visualized graphically along two dimensions. PCA of the athletic performance indicators was conducted using FactoMineR and factoextra R packages, and the data was clustered (unsupervised learning). The validity of the clustering results was assessed to determine whether they successfully distinguished between the sports of the samples. Since there were 18 subjects, aggregate hierarchical clustering was performed [29,30].

## 3. Results

The results of the tennis talent identification study were analyzed into two parts: indicator analysis and clustering results. Players with tennis talent were successfully identified with the tennis talent identification method developed in this study. It can be seen that exercises for both explosive power and flexibility are necessary for tennis players. For example, subject tennis player 1 is at the center of the tennis player cluster. Moreover, there is a significant difference in explosiveness and flexibility between subjects who specialize in tennis and those who specialize in basketball and between males and females. The explosive power of soft tennis players was significantly lower than that of tennis players and basketball players, and the flexibility of basketball players was significantly lower than that of tennis players. The analysis of the results are described below.

### 3.1. Indicator Analysis

**Figure 1:**
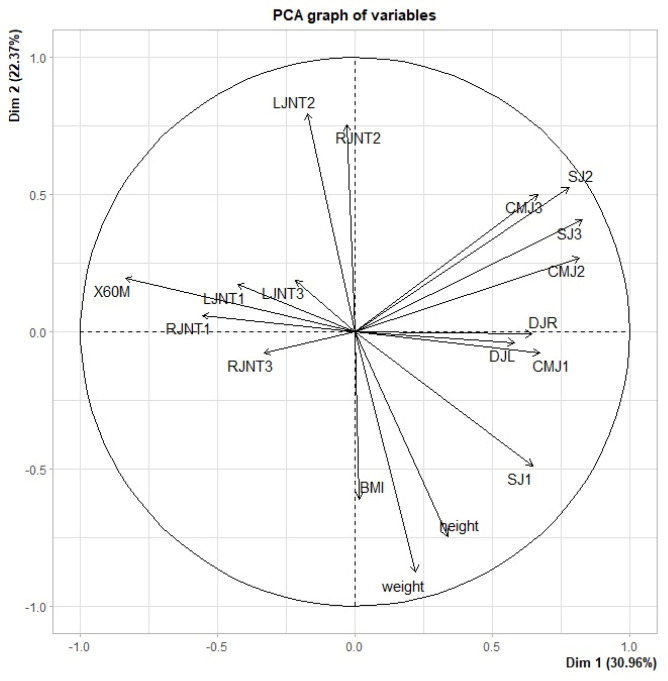
Principal component analysis results with indicator distribution.

A total of 18 test indicators, which comprise of 15 indicators mentioned in the aforementioned literature as well as the height, weight, and BMI of the subjects, were used to distinguish between the respective sports of the samples. Scatter plots to describe the relationship between samples were not possible, due to a large number of indicators; therefore, PCA was performed to reduce the dimensionality of the large data set. The PCA results for the performance of the 18 samples in the 18 indicators are shown in Figure 1 [29,30].

According to Figure 1, the indicators most representative of Dim1 are SJ 3, CMJ 2, and 60 M. SJ 3, CMJ 2, SJ 2, CMJ 3, SJ 1, DJR, DJL, RJNT 1, and 60 M were significantly correlated to Dim1. These indicators, which have been discussed in the aforementioned literature, all measure the explosive power of the samples; thus, Dim1 represents explosive power. Furthermore, 60 M is an example of an indicator that is negatively correlated with Dim1; the longer the subject takes to do the 60 m shuttle run, the larger the indicator value is, indicating less explosiveness. The correlation results between the indicators significantly correlated to Dim1 are presented in Table 1.

According to Figure 1, that indicators LJNT 2 and RJNT 2 are the most representative of Dim2. Table 2 reveals that LJNT 2, RJNT 2, SJ 2, CMJ 3, and the height and weight of the samples were significantly correlated to Dim2. The indicators above measure flexibility, as discussed in the aforementioned literature, thus, Dim2 represents flexibility. Height and weight have significant negative correlations with flexibility.

According to the results shown in Table 1 and Table 2, most of the indicators can be used to measure explosive power and flexibility. Only a few indicators, such as LJNT 1, RJN 3, LJN 3, and BMI, did not show significant correlations with explosive force and flexibility. However, it can be seen that SJ 2 and CMJ 3 can measure both the explosive power and flexibility of the athletes.

### 3.2. Clustering Results

**Figure 2:**
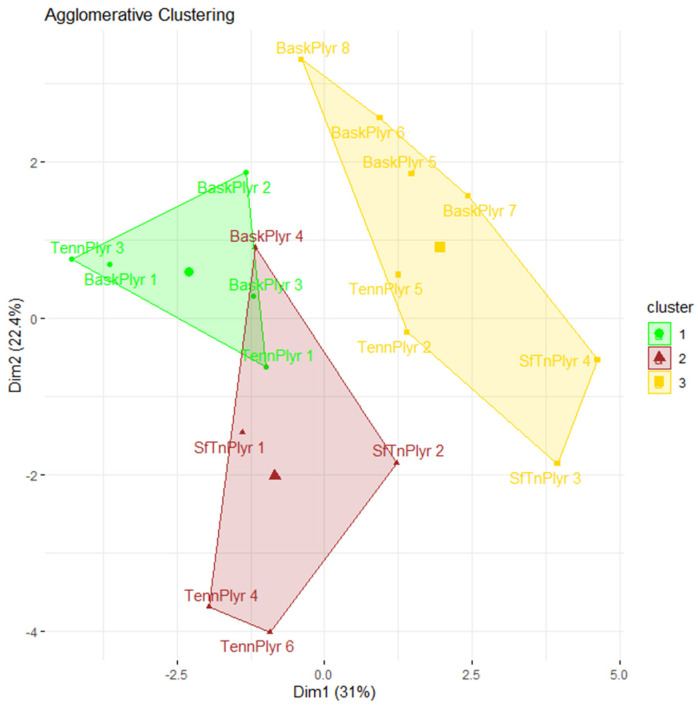
Aggregate hierarchical clustering results for the performance of 18 samples.

As shown in Figure 2 and Table 3, the first group (green) was composed of six samples, and the coordinates of the centroid were (−2.4, 0.8); the second group (red) was composed of four subjects, and the centroid was at (−0.3, −2); the third group (yellow) consisted of eight samples, and its centroid coordinates were (2.3, 0.9). Figure 2 shows that after agglomerative clustering was performed, the native groups of basketball, tennis and soft tennis players are clustered into the new three clusters of “strength” by Cluster 1, 2 and 3 (Figure 2). For example, Cluster 3 (yellow) has the largest number of tennis players and it indicates the strongest tennis “strength”. Thereafter, Cluster 1 and Cluster 2 are to be interpreted similarly.

**Figure 3:**
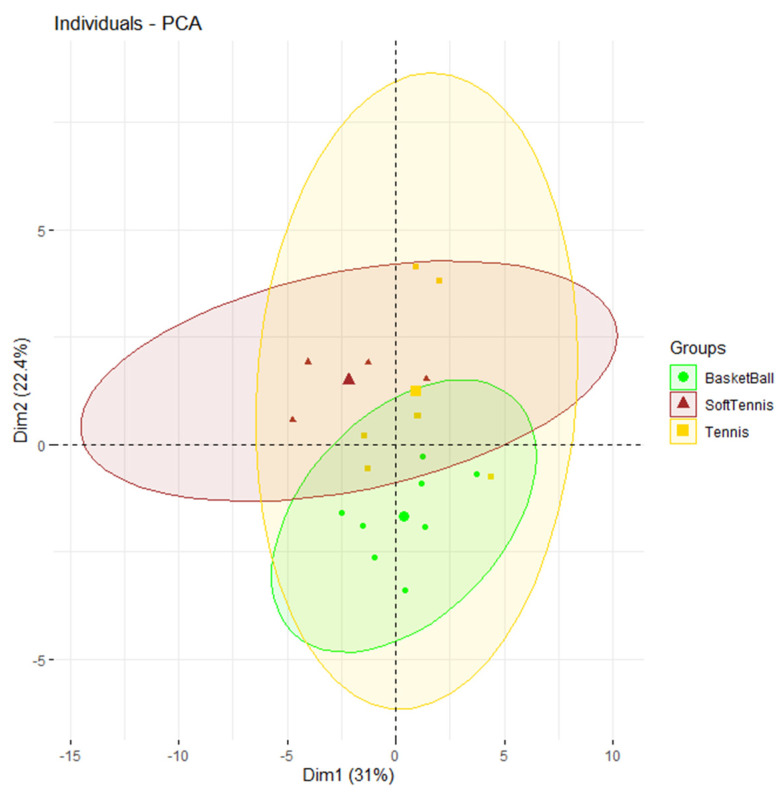
Confidence interval estimate for each group.

The clustering results were further analyzed using FactoMineR and factoextra to calculate the confidence interval for the average of each group [30]. Figure 3 shows there is no overlap between the confidence ellipse of each group, indicating that there are significant differences in explosive power and flexibility of the sports. The results indicated that the explosive power of the soft tennis players was significantly lower than that of the tennis and basketball players; in addition, the flexibility of the third group, composed of the basketball samples was significantly lower than that of the tennis and soft tennis players. Even though FactoMineR does not display less significant items between groups, it can be seen in Figure 3 that the confidence intervals of the tennis and basketball samples overlapped in Dim1, i.e., explosive power. However, the two groups are separated on the plot due to the significant difference between them in Dim2 (flexibility). 

In Dim1, the confidence interval of the soft tennis players does not overlap with that of the other groups, indicating that the explosive power of the soft tennis players was significantly lower than that of the tennis and basketball samples. Comparing the tennis and soft tennis players, there was only a significant difference in explosive power; there was no significant difference in flexibility between genders. There was no significant difference in terms of explosive power between the basketball and tennis groups, though there was a significant difference in flexibility.

## 4. Discussion

This study found that the right shoulder joint internal rotation (RJNT 1) was significantly correlated to explosive power. In tennis, the serve and forehand involve right shoulder internal rotation. Sufficient shoulder mobility means that the player has more space to take the racket back and accelerate it before hitting the ball, which generates more power. Serves and forehand shots are essential to a tennis player’s offensive game; therefore, shoulder mobility is an important indicator for tennis talent identification.

This study is the first domestic application of sports talent classification assessing the vertical jump, shuttle run, and shoulder mobility to determine whether the subjects are suitable for their sport. The results showed that there were significant differences between men’s tennis, women’s tennis, and men’s basketball in the two dimensions. In particular, this classification method has great implications for evaluating tennis talent among male tennis players. For example, the tennis subject tennis player 1 is a National Junior Ranking Tournament Level A singles champion and a team event champion in the National High School Games. His scores can be regarded as evidence of tennis talent, and tennis player 1 is also at the center of the yellow ellipse in Figure 3, which shows that his performance in these two dimensions possesses a special combination of characteristics that indicate tennis talent. Another example is shown by the clustering of the samples who specialize in basketball. The soft tennis region is different in that it contains certain basketball players who were slower. 

The coordinates of basketball player 3 is in the tennis player region. After checking the height and weight (176 cm, 81 kg) of basketball player 3, it was found that the player may be more suitable for tennis. Tennis player 3 possesses more explosive power than any other tennis player but is at a disadvantage in terms of flexibility, resulting in a performance plateau that needs to be overcome.

Tennis and basketball defender’s manifest explosive power in similar ways, such as sudden changes in direction (forward, backward, left, and right), abrupt stop/start movements, and jumping. Upon closer examination, tennis can be said to be a sport involving multidirectional shuttle runs. During a sudden start and stop movement, the torso must rotate with the swing of the upper limbs, and the upper limbs must coordinate the racket swing with the lower limbs to hit the ball. In other words, each sprint has a purpose, and a tennis player must repeatedly execute a continuous pattern—sprint, abrupt stop, and swing. Fernandez-Fernandez, Sanz-Rivas, and Mendez-Villanueva (2009) [24]; Kovacs (2006) [25]; and Chiang, Tsai, and Chiang (2015) [20] pointed out that in a tennis match, the duration of a point is between 2 and 10 s, the number of directional changes in a point is about 3–4, and the number of strokes is about 4–5. Moreover, athletes run at high intensity to hit each ball, and the running distance depends on the player’s age, level, and court surface.

Although basketball players also have sudden directional changes during a game, sometimes after having to abruptly stop to make a jump shot or after a sprint, there will be a natural recovery period. In contrast, following a serve, tennis players must continuously sprint in various directions. One similar aspect is that basketball and tennis players use the upper limbs to complete all shots, but without the lower limbs supporting the entire body’s center of gravity and maintaining balance, players cannot perform well on the court [22].

The jumps tested in this study are indicative of a player’s explosive power, which is most apparent in the tennis serve, basketball jump shot, and basketball rebound because they all require a burst of lower body strength to jump, extend the entire body, and hit, shoot, or retrieve the ball at the highest point of the jump. At the same time, the vertical jump test also provides insight into a subject’s reaction time and movement initiation time.

In this study, the shuttle run test required subjects to run back and forth every 20 m three times. As the subjects repeatedly sprint, decelerate, and accelerate, their explosive power can be evaluated, and as the subjects decelerate to touch the cone and turn, their flexibility can be observed. Athletes with better flexibility can extend their bodies further to touch the cone. This phenomenon is shown in Figure 1 and Figure 2 with tennis player 1 and tennis player 3. Tennis player 1 is a National Junior Ranking Tournament Level A singles champion and a team event champion in the National High School Games, and the highest achievement of tennis player 3 is placing top eight in the National High School Games team event. The two players have a similar build, style of play, and number of years of experience. The greater flexibility of tennis player 1 allows for more variation in hitting movements, which requires full-body coordination, and allows him to execute advanced shots on the court in critical moments.

Tennis players need shoulder mobility to take the racket back to prepare for a shot after each sudden start and stop movement, and the muscles around the shoulder joint are also important for executing the swing to hit the ball [22]. Passive and active structures for joint stability and the neuromuscular control system are responsible for shoulder joint stabilization in humans [31]. Tennis is an overhead sport, and the shoulder joint, as a ball-and-socket joint, allows for a greater range of motion. The range of motion for shoulder flexion and abduction is 180 degrees, and it is 90 degrees for internal/external rotation. The joint capsule and ligaments surround and stabilize the shoulder joint throughout its wide range of motion [26]. This study found that elite tennis players had better shoulder mobility, which was particularly evident in their serves. When serving, full external rotation of the shoulder joint allows more room for the racket to accelerate in the acceleration phase, which can further increase power upon contact with the ball. In addition, players with greater shoulder mobility can also execute various types of serves. The flat serve, topspin, and slice serve are more common types of serves, but for a kick serve, shoulder mobility is necessary to achieve external rotation to hit the ball in a way that makes it difficult for the receiving player to return the serve.

## 5. Conclusions

In this paper, we mainly discussed the identification of natural athletic talent. Outstanding athletic performance can be a consequence of genetic inheritance or acquired learning; thus, it is important to identify athletes with the highest potential for success. This study used the talent identification and testing methods developed by a strength and conditioning coach to scientifically distinguish the differences between the physical attributes of tennis and basketball players. The tennis talent testing system developed in this study can provide advice to young players who intend to pursue careers in professional tennis. For example, if an athlete’s explosive power and joint flexibility are considered not suitable for professional tennis, the athlete, parents, and coaches would be advised accordingly, i.e., the athlete would be advised against pursuing a professional career in tennis. Therefore, it is imperative to understand whether an athlete’s physical attributes are suitable for training and competing in a particular sport.

## Figures and Tables

**Table 1:** Indicators significantly correlated to Dim1.

Dim1	Correlation	*p*. Value
SJ 3	0.8281	*p* < 0.0001
CMJ 2	0.8179	*p* < 0.0001
SJ 2	0.7781	*p* < 0.0001
CMJ 1	0.672	0.0023
CMJ 3	0.6644	0.0026
SJ 1	0.6484	0.0036
DJR	0.6448	0.0039
DJL	0.5815	0.0114
Height	0.3368	0.1718
Weight	0.222	0.3756
BMI	0.0161	0.9496
RJNT 2	−0.0308	0.9034
LJNT 2	−0.1746	0.4883
LJNT 3	−0.2159	0.3895
RJNT 3	−0.3322	0.1780
LJNT 1	−0.4287	0.0759
RJNT 1	−0.5564	0.0165
60 M	−0.8369	*p* < 0.0001

**Table 2:** Indicators significantly correlated to Dim2.

Dim2	Correlation	*p*. Value
LJNT 2	0.7955	*p* < 0.001
RJNT 2	0.7551	0.0003
SJ 2	0.5269	0.0247
CMJ 3	0.5018	0.0338
CMJ 2	0.2693	0.2799
60M	0.1956	0.4367
LJNT 3	0.1877	0.4559
LJNT 1	0.1717	0.4958
RJNT 1	0.0599	0.8134
DJR	−0.0081	0.9746
DJL	−0.0388	0.8784
RJNT 3	−0.0769	0.7617
CMJ 1	−0.0773	0.7604
SJ 1	−0.4892	0.0394
BMI	−0.6092	0.0073
height	−0.7465	0.0003
weight	−0.8768	*p* < 0.001

**Table 3:** Comparison of hierarchical clustering results.

Hierarchical Subgroup		Group 1	Group 2	Group 3
Number of athletes	Tennis	6		
	Soft Tennis		4	
	Basketball			8

## Data Availability

All data are available upon reasonable request to the corresponding author.
